# Depression, Anxiety, and Stress in Kazakhstani Women with Recurrent Pregnancy Loss: A Case–Control Study

**DOI:** 10.3390/jcm12020658

**Published:** 2023-01-13

**Authors:** Assylzhan Issakhanova, Alpamys Issanov, Talshyn Ukybassova, Lyazzat Kaldygulova, Aizada Marat, Balkenzhe Imankulova, Nazira Kamzayeva, Wassim Y. Almawi, Gulzhanat Aimagambetova

**Affiliations:** 1 School of Medicine, Nazarbayev University, Astana 010000, Kazakhstan; 2 Department of Medicine, School of Medicine, Nazarbayev University, Astana 010000, Kazakhstan; 3 School of Population and Public Health, University of British Columbia, Vancouver, BC V6T 1Z4, Canada; 4 Clinical Academic Department of Women’s Health, Corporate Fund “University Medical Center”, Astana 010000, Kazakhstan; 5 Department of Obstetrics and Gynecology #2, West-Kazakhstan Marat Ospanov Medical University, Aktobe 030012, Kazakhstan; 6 Department of Obstetrics and Gynecology #1, NJSC “Astana Medical University”, Astana 010000, Kazakhstan; 7 Department of Biomedical Sciences, School of Medicine, Nazarbayev University, Astana 010000, Kazakhstan; 8 Faculte’ des Sciences de Tunis, Universite’ de Tunis El Manar, Tunis 1068, Tunisia

**Keywords:** recurrent pregnancy loss, stress, anxiety, depression, Kazakhstan

## Abstract

Background: Recurrent pregnancy loss (RPL) is associated with increased incidence and severity of depression, anxiety, and stress, and screening for these comorbidities following miscarriages is beneficial for women with RPL who are planning future pregnancies. This study aims to investigate depression, anxiety, and stress among Kazakhstani women with RPL. Methods: This was a case–control study involving 70 women with confirmed RPL and 78 ethnically matched control women. Depression, anxiety, and stress were evaluated using the Depression Anxiety Stress Scales (DASS)-21 instrument. Linear regression and correlation analysis were used in assessing the association of RPL with symptoms of depression, and/or anxiety, and/or stress, after adjusting for key covariates. Results: Women with RPL were found to have significantly higher mean scores for depression (*p* < 0.001), anxiety (*p* < 0.001), and stress (*p* < 0.001) symptoms. Mild–moderate stress and mild–moderate and severe–extreme depression and anxiety symptoms were more frequent in the RPL group than in the control group. Regression analysis demonstrated that RPL was the only significant variable associated with anxiety, depression, and stress symptoms. Conclusion: The results of this study suggest that women with RPL are more likely to experience heightened symptoms of depression, anxiety, and stress. Proper psychological counseling is recommended for women with RPL, as well as their spouses.

## 1. Introduction

Recurrent pregnancy loss (RPL) is a reproductive health issue that affects approximately 1–2% of otherwise healthy women worldwide [1]. RPL is defined by the World Health Organization (WHO) as three or more consecutive losses of fetuses up to 20 weeks of gestation [2]. Other definitions were also suggested, including that of the Royal College of Obstetricians and Gynecologists (RCOG), which defines RPL as three or more consecutive pregnancy losses before 24 weeks of gestation [3], and the American Society of Reproductive Medicine (ASRM), in which RPL diagnosis is confirmed as two or more clinical pregnancy losses, which are not necessarily consecutive [4]. More recently, the European Society of Human Reproduction and Embryology (ESHRE) revised the definition of RPL to include two or more pregnancy losses confirmed by serum or urine human chorionic gonadotropin and excluding confirmed ectopic or molar pregnancies [1]. 

RPL is a complex multifactorial disorder, and various etiological causes contribute to its pathogenesis. These include genetic [5,6], anatomic abnormalities, endocrine disorders, infections [7,8], thrombophilia (heritable or acquired) [9,10], and environmental factors [3,11,12,13,14]. However, in nearly half of women with RPL, the definitive cause remains unexplained [3,13,15,16,17,18]. Increasing evidence indicated that idiopathic RPL is linked with marked emotional stress specific to pregnancy failure, thus posing additional management needs for women with RPL [19,20].

The unintentional loss of a desired pregnancy is a distressing life event and is accompanied by an array of psychological comorbidities, including grief, fear, anxiety, stress, relational conflict, marital distress, and poor personal adjustment [14,21]. Studies from different ethnic groups converge on the link between psychological impairments and RPL [19,22,23,24], highlighted by the five times higher risk of moderate/severe depression symptoms in women with idiopathic RPL compared to women with an uncomplicated pregnancy and delivery [24]. It was suggested that untreated chronic stress and depression may induce the activation of the physiologic stress response linked with increased cortisol levels, which in turn alters the helper T cell (Th)1-Th2 balance required for the maintenance of pregnancy [10,25], resulting in accelerated miscarriages and a vicious cycle for women with RPL [26]. Collectively, this emphasizes the need for careful assessment of psychological comorbidities in RPL and eventually devising counseling strategies for women with a history of RPL who are considering future pregnancy. 

Maternal and reproductive health is managed by the Ministry of Healthcare (MoH) in the Republic of Kazakhstan, and special programs were developed to support reproductive health and improve its outcomes [27,28]. Considering that the traditional family structure in Kazakhstan supports and promotes parenthood, the evaluation of reproductive health indicators significantly affects the quality of life [28]. Earlier statistics based on regional reports suggest that infertility is diagnosed in 9% and perinatal losses in 30% of reproductive-age women in the western and southern regions of Kazakhstan [29]. Despite these figures, there is a lack of reliable epidemiological data on the incidence of RPL in Kazakhstan, and almost no indicator of the extent of symptoms of stress, depression, and anxiety among women with RPL. This study evaluates depression, stress, and anxiety symptoms in women with RPL compared to women with no history of RPL, both of whom attended an outpatient clinic in Astana, Kazakhstan. This will help to fill the information gap and provide the basis for a holistic approach to RPL management.

## 2. Materials and Methods

### 2.1. Study Subjects and Recruitment

This is a case–control study involving 70 women with documented RPL based on ESHRE definition of two or more pregnancy losses (age, 18–40 years). In addition, 78 women with a history of two or more uncomplicated pregnancies with either vaginal delivery or cesarean section and no history of RPL were included as a control. Recruitment of RPL cases and control women took place during a routine visit at the outpatient gynecology clinic of the University Medical Center (UMC), National Research Center for Mother and Child Health (NRCMCH), Astana, Kazakhstan. The NRCMCH is a tertiary care hospital, which provides obstetrics and gynecology care for women referred from the whole country. The gynecology outpatient clinic of the hospital has on average 2000 outpatient visits per month and provides a full range of obstetrics and gynecology care. Inclusion criteria for RPL included reproductive age (18–40 years old), two or more miscarriages, and the ability to answer survey questions. Exclusion criteria for both groups included older age (>40 years) at first pregnancy, preclinical miscarriages, and/or biochemical pregnancy, severe psychiatric disorders, drug therapies that affect mood, and clinical conditions such as hyper- or hypothyroidism. Patients were also excluded if they underwent controlled ovarian hyperstimulation and artificial insemination (COH/AIH), since they may potentially affect the main findings. There was no limitation in patient recruitment based on the time of RPL occurrence. 

### 2.2. Study Instrument

Demographic and clinical data from RPL cases and control women were collected using a standardized questionnaire. Collected data included age, ethnicity, height and weight, education, income, menarche, menstrual function, and history of gynecological illnesses. Personal and family history of RPL, along with the number of pregnancies, live births, abortions (and type), gestational age at the time of abortion, and previous infertility treatment were collected from all participants. Furthermore, factors associated with miscarriage, including Papanicolaou test (Pap test), infections (and type), previous use of contraceptives, smoking (none, current, past), alcohol intake, and chronic non-gynecological illnesses were also obtained from RPL cases and control women. 

The levels of depression, anxiety, and stress were assessed using the Depression, Anxiety, and Stress Scale -21 Items (DASS-21) instrument [30,31], which consists of 3 subscales with 7 items in each assessing participant’s emotional state as experienced over the past week. By responding to every item on a 4-point severity scale (0, does not apply to me at all; 1, applies sometimes; 2, applies to a considerable degree; and 3, applies most or all the time) the final scores of depression, anxiety, and stress were summed up and categorized into normal, mild–moderate, and severe [30,31,32].

#### Assessment of the Instrument

The internal reliability and validity of the constructs (stress, anxiety, and depression) on the study population were also assessed. The internal reliability was examined using Cronbach’s alphas and their corresponding 95% confidence intervals (CIs), calculated using bootstrapping 1000 samples, and the corrected item-total correlation, using Pearson’s correlation coefficient estimation. The following cut-offs were used in deciding whether corrected item-total correlations were adequate: less than 0.20—inadequate, 0.20–0.70—adequate, and above 0.70—redundant [33]. DASS-21 and the unified study questionnaire were made available in three validated versions, English, Russian, and Kazakh for study participants.

To assess whether the DASS-21 scale structure fitted the study population data (whether items within each subscale were appropriately placed within their corresponding constructs), a confirmatory factor analysis (CFA) with the quasi-maximum likelihood estimation approach was performed. First, CFA assumptions (the normality, inter-item correlations, and response scale format) were checked and then, when the assumptions were met, we proceeded to conducting the analysis. We used the cut-off 0.3 or above in absolute value as the indication of sufficient factor loadings (sufficient evidence of a correlation between an item and factor (construct) to suggest that the item loads on the factor (construct)). Next, the cut-off 0.8 or higher in error variance indicating a poor item loading was used. Additionally, we used root mean square error of approximation (RMSEA), comparative fit index (CFI), and standardized root mean square (SRMR) estimators when assessing the goodness of fit of the factor (construct) structure. The following cut-offs were used when assessing a goodness-of-fit level: the upper bound of 90% for RMSEA is 0.10 or lower—adequate fit, RMSEA of 0.08 or lower—excellent fit; CFI of 0.90 or above—fair fit, CFI of 0.95 or above—excellent fit; and SRMR value of 0.08 or lower—adequate fit, SRMR of 0.05 or lower—excellent fit. 

To examine the validity of the subscales (whether items within the subscales measured what they were intended to), we used correlation with “other criterion” known to be associated based on theory or empirical data [34]. We tested correlations between stress, anxiety, and depression constructs using Pearson’s correlation coefficient calculations, as theoretical frameworks and empirical data suggested the existence of strong positive correlations between these constructs [35,36,37,38,39,40].

### 2.3. Ethical Considerations

Following a standard face-to-face appointment in a quiet office after explaining the study aims and signing the informed consent, participants were asked to answer the 21 questions included in the DASS-21 instrument. All study subjects (cases and controls) were guaranteed anonymity during data processing and informed written consent was required for participation in the study. The study was approved by the Nazarbayev University School of Medicine Institutional Research Ethics Committee (NUSOM-IREC-JUNE-2020-#01; granted on 20 August 2020) and UMC IRB (protocol #2, granted on 30 June 2020). 

### 2.4. Statistical Analysis

Statistical analysis was performed using STATA and R statistical software [41,42]. Descriptive statistics (frequencies, mean, standard deviation, percentages) were used to present the data; χ2 tests (or Fisher’s exact tests for low numbers) were performed to compare relative frequency distributions of categorical variables between control and cases (having history of two or more pregnancy losses). Student’s *t*-tests or Mann–Whitney U-test, where the parametric test assumptions were not satisfied, were utilized to assess differences of means in continuous variables of controls and cases. Separate multivariable linear regression models (causal modelling approach) were built to assess the associations of a history of RPL (the primary exposure variable) with stress, anxiety, and depression as the continuous outcomes. Based on literature review and clinical importance of patient characteristics in relation to the patient RPL and psychological characteristics, age, BMI, education, and ethnicity were included in the models as potential confounding covariates [43,44,45]. A *p* < 0.05 was considered statistically significant.

## 3. Results

### 3.1. Study Population

The socio-demographic and clinical characteristics of the 78 control women and 70 women with RPL are presented in Table 1. Age (*p* = 0.07), BMI (*p* = 0.91), family income (*p* = 0.51), along with the age of menarche (*p* = 0.22) and the frequency of gynecological illnesses (*p* = 0.54) were not statistically different between RPL cases and control women. Increased frequency of low-level education was found in the control group, while university-level education was more prevalent in RPL cases (*p* = 0.009). Compared to control women, a significantly higher number of pregnancies (*p* < 0.001) and lower number of live births (*p* < 0.001) was noted in RPL cases.

### 3.2. Assessment of the Instrument

The Cronbach’s alpha for the stress construct was 0.89 (95% CI: 0.86; 0.92), for the anxiety construct was 0.86 (95% CI: 0.80; 0.89), and for the depression construct was 0.88 (95% CI: 0.83; 0.92). Item-total correlations without an item (corrected item-total correlations) ranged for the stress construct between 0.66 and 0.74, for the anxiety construct between 0.48–0.70, and for the depression construct 0.59–0.75, indicating that the included items appropriately discriminated their corresponding constructs.

In CFA, all items loaded on the factors (constructs—stress, anxiety, and depression), factor loadings ranging from 0.64 to 0.89. Error variances for the items were acceptable (lower than 0.80), ranging between 0.29 and 0.59. We also examined the goodness of fit of the factor structure of the questionnaire. The CFI estimate indicated the excellent fit (0.973). Both the upper bound of 90% CI for RMSEA was on the borderline 0.08 and the estimate of SRMR was 0.063, suggested adequate fit of the factor (construct) structure of the questionnaire. Lastly, we assessed correlations between the constructs to check the validity of the subscales using “the correlation with “other criterion” known to be associated based on theoretical framework or empirical data” approach. The Pearson correlation coefficient between stress and anxiety was 0.84, between stress and depression was 0.84, and between anxiety and depression was 0.82.

### 3.3. Prevalence of Symptoms of Depression, Anxiety, and Stress

The prevalence of depression, anxiety, and stress symptoms in the RPL group and control group are presented in Table 2. 

### 3.4. Regression Analysis

Regression analyses for symptoms of stress, anxiety, and depression are demonstrated in Table 3. 

Covariates for stress, anxiety, and depression symptoms were introduced both as continuous (age) and categorical variables (ethnicity, education). History of RPL was positively associated with higher levels of stress (*p* < 0.001), anxiety (*p* < 0.001), and depression (*p* < 0.001) symptoms, after adjusting for age, ethnicity, BMI, and education. Other variables such as age, BMI, education status, and ethnicity were not predictive of the levels of depression, anxiety, and stress symptoms (*p* > 0.05).

## 4. Discussion

Recurrent miscarriage is a major reproductive health issue linked with risks of gynecologic complications and long-term health problems extending beyond pregnancy [14]. Psychological comorbidities, including depression, anxiety, and stress, are frequently linked with increased risks of pregnancy loss [14,24]. However, the impact of psychological distress on patients’ health and its contribution to the rates of subsequent miscarriages and overall reproductive morbidity is underestimated. Our results demonstrated that women with RPL are more likely to experience symptoms of depression, anxiety, and stress. This is the first study that addresses the link between RPL and depression, anxiety, and stress in Kazakhstan and neighboring former Soviet Union central Asian republics, and the results obtained prompt recommendations for the inclusion of psychological counseling for women with RPL and their spouses in the management of RPL.

The choice of DASS-21 as the screening instrument was based on its confirmed usefulness in measuring depression, anxiety, and stress in one visit. We validated the utility of DASS-21 by comparing the anxiety and depression scores generated from the 21-item DASS-21 with the 14-item Hospital Anxiety and Depression Scale (HADS) in type 2 diabetes [31] and sickle cell anemia [46], whereby participants were asked to fill both DASS-21 and HADS in the same session. Correlation between both instruments, assessed by Spearman r2 correlation, was high between DASS-21 and HADS in assessing depression and anxiety, and was not affected by differences in gender, age distribution, and socioeconomic status [31,46].

Depression, anxiety, and stress symptoms were positively correlated with RPL, and their mean scores were significantly higher among women with confirmed RPL. The results of this study were reminiscent of the finding of Wang et al. (2021), which reported a higher prevalence of depression and anxiety among women with RPL [47], suggesting that existing symptoms of depression and anxiety are contributors to the increased risk of RPL. A similar outcome was reported in studies on Chinese [48], Danish [49], and Iranian [50] populations. 

However, our study did not note any association between the level of education, household income, residence (rural, urban), and the three tested psychological comorbidities (depression, anxiety, and stress). In comparison, a recent Chinese study identified low household income, low level of education, history of miscarriages, and no live births to be associated with higher levels of depression and anxiety symptoms in women with RPL [48]. This was in line with an earlier study, which demonstrated a lower degree of depression and anxiety in women with RPL having at least one live birth compared with RPL cases with no live birth [50]. We attribute this apparent contradiction between our findings and those published elsewhere to the relatively small sample size, and to the ethnic background of study participants [51,52]. 

Similar results as were reported in Danish women with RPL, where the prevalence of symptoms of depression, anxiety, and stress was higher in RPL cases, and interestingly among male partners of women with RPL [49]. While not reported here, a parallel trend for worsening depression and stress was also noted for spouses of Kazakhstani RPL cases. It is tempting to speculate that spouses of women with RPL become more strained seeing their wives developing depressive, irritable, or grieving moods [53], whereby men become more responsible for their partner’s well-being, and generally maintain a positive and supportive attitude towards their partners while experiencing the feelings of loss linked with RPL [49,53]. Future population-based case–control study in Kazakhstan is warranted, considering the ethnic and cultural differences in the country [54,55].

Clinical implications. By confirming significant levels of stress, depression, and anxiety symptoms among women with RPL, this study underscores the importance of screening for psychological morbidity among women with RPL. Women diagnosed with RPL should receive preconception counseling and care, including psychological support. In particular, early psychological support should be initiated for those who are at high risk of psychological distress as a part of their infertility treatment.

Strengths and limitations. Our study has several strengths, namely that it was the first study to investigate stress, depression, and anxiety among Kazakhstani women with RPL, that RPL cases and control women were matched according to their ethnicity, and that the diagnosis of RPL was confirmed by medical records and not self-reported. However, the study had a number of shortcomings, mostly in the relatively small number of participants (its main limitation), and in its (case–control) study design, which prevented estimation of the cause–effect relationship between RPL development and symptoms of depression, anxiety, and stress. Another shortcoming was the lack of psychological history of anxiety or depressive disorders, and treatment for these and related disorders, both of which are predictors for subsequent episodes of illness. Assessing pre-pregnancy psychological profile is very subjective, and is influenced by personal, socioeconomic, and societal norms and beliefs, as a society may not recognize the impact of this loss for the parents. Most mothers are at high risk of anxiety and depression [56], which are higher during pregnancy than during other life periods. This intensifies in vulnerable populations, including women with RPL [57], since this demands adaptations to an abrupt and evolving situation, which triggers psychological changes in future pregnancies. Future, prospective cohort studies with a higher number of participants are needed to provide a clearer picture of the topic of RPL in Kazakhstan and explore the causal relationship between RPL development and depression, anxiety, and stress.

## 5. Conclusions

Psychological morbidity is common among women with pregnancy loss. The results of our study demonstrate that Kazakhstani women with a history of recurrent pregnancy loss have higher levels of stress, depression, and anxiety in comparison with women who had successful pregnancy outcomes. Effective screening instruments and management options for psychological consequences should be available for women after miscarriage. 

## Figures and Tables

**Table 1:** Socio-demographic, reproductive characteristics and medical history of the study participants attended the National Research Center for Mother and Child Health, Astana, Kazakhstan.

Variable	Total (*n* = 178)	Controls (*n* = 78)	RPL Cases (*n* = 70)	t/χ2	*p*-Value
Age in years, mean ± SD	34.8 ± 7.1	35.7 ± 7.0	33.6 ± 7.0	t = 1.82	0.07
BMI in kg/m^2^, mean ± SD	24.5 ± 4.1	24.6 ± 4.2	24.5 ± 4.1	t = 0.15	0.91
BMI categories, *n* (%)				χ^2^ = 0.01	0.55
<25 kg/m^2^	90 (60.8) ^3^	47 (60.3)	43 (61.4)		
≥25 kg/m^2^	57 (38.5)	31 (39.7)	26 (37.1)		
Ethnicity, others *n* (%)	13 (8.8)	5 (6.4)	8 (11.4)	χ^2^ = 0.62	0.43
Education, *n* (%)				χ^2^ = 9.62	0.009
Elementary	31 (20.9)	24 (30.8)	7 (10.0)		
Secondary	21 (14.2)	11 (14.1)	10 (14.3)		
University	94 (63.5)	43 (55.1)	51 (72.9)		
Family income, *n* (%)				χ^2^ = 1.18	0.51
Low	29 (19.6)	18 (23.1)	11 (15.7)		
Middle	108 (73.0)	55 (70.5)	53 (75.7)		
High	10 (6.8)	5 (6.4)	5 (7.1)		
Menarche in years, mean ± SD	13.5 ± 1.2	13.6 ± 1.2	13.4 ± 1.2	t = 1.23	0.22
Gynecological illnesses, *n* (%)	25 (16.9)	14 (17.9)	11 (15.7)	χ^2^ = 0.01	0.54
Number of pregnancies, mean ± SD	3.8 ± 1.8	3.3 ± 1.5	4.5 ± 1.9	t = −4.37	<0.001
Para, mean ± SD	1.7 ± 1.2	2.4 ± 0.9	0.9 ± 1.1	t= 9.14	<0.001
Medical history					
Toxoplasma gondii, *n* (%)	23 (15.5)	12 (15.4)	11 (15.7)	χ^2^ = 2.37	0.20
HCMV, *n* (%)	23 (15.5)	13 (16.7)	10 (14.3)	χ^2^ = 2.77	0.17
Chlamydia trachomatis, *n* (%)	22 (14.9)	12 (15.4)	10 (14.3)	χ^2^ = 3.40	0.13
Rubella, *n* (%)	25 (16.9)	12 (15.4)	13 (18.6)	χ^2^ = 2.70	0.28
Ureaplasma, *n* (%)	25 (16.9)	12 (15.4)	13 (18.6)	χ^2^ = 11.98	0.003
Previous oral contraceptives, *n* (%)	23 (15.5)	15 (19.2)	8 (11.4)	χ^2^ = 1.02	0.15
Smoking, *n* (%)	6 (4.1)	3 (3.8)	3 (4.3)	χ^2^ = 0.00	1.00
Alcohol, *n* (%)	5 (3.4)	4 (5.1)	1 (1.4)	χ^2^ = 0.55	0.09
History of a chronic disease, *n* (%)	12 (8.1)	5 (6.4)	7 (10.0)	χ^2^ = 0.25	0.62
Family history of a chronic disease, *n* (%)	10 (6.8)	5 (6.4)	5 (7.1)	χ^2^ = 0.00	1.00
Hypertension, *n* (%)	7 (4.7)	3 (3.8)	4 (5.7)	χ^2^ = 0.02	0.88
Thyroid disease, *n* (%)	16 (10.8)	5 (6.4)	5 (7.1)	χ^2^ = 0.24	0.62

Medical history characteristics of the study participants are presented in Table 1. Apart from *Ureaplasma* infection which was higher in RPL cases than in control women (18.6% vs. 15.3%; *p* = 0.003), no statistically significant differences were found between RPL cases and control subjects.

**Table 2:** Stress, depression, and anxiety levels among the study participants attended the National Research Center for Mother and Child Health, Astana, Kazakhstan.

Variable	Total (*n* = 148)	Controls (*n* = 78)	RPL Cases (*n* = 70)	t/χ2	*p*-Value
DASS-21 stress score continuous, mean ± SD	6.46 ± 5.11	4.83 ± 4.60	8.27 ± 5.07	t = −4.30	<0.001
DASS-21 stress severity, *n* (%)				χ^2^ = 3.58	0.773
Normal	135 (91.2)	72 (92.3)	63 (90.0)		
Mild–moderate	13 (8.8)	6 (7.7)	7 (10.0)		
DASS-21 anxiety score continuous, mean ± SD	4.33 ± 4.48	2.74 ± 3.30	6.10 ± 4.95	t = −4.80	<0.001
DASS-21 anxiety severity, *n* (%)				χ^2^ = 11.91	0.003
Normal	119 (80.4)	70 (89.7)	49 (70.0)		
Mild–moderate	23 (15.5)	8 (10.3)	15 (21.4)		
Severe–extreme	6 (4.1)	0 (0.0)	6 (8.6)		
DASS-21 depression score continuous, mean ± SD	4.58 ± 4.83	2.92 ± 3.37	6.43 ± 5.52	t = −4.60	<0.001
DASS-21 depression severity, *n* (%)				χ^2^ = 15.55	<0.001
Normal	127 (85.8)	75 (96.2)	52 (74.3)		
Mild–moderate	20 (13.5)	3 (3.8)	17 (24.3)		
Severe–extreme	1 (0.7)	0 (0.0)	1 (1.4)		

Compared to control subjects, women with RPL had significantly higher scores of depression (*p* < 0.001), anxiety (*p* < 0.001), and stress (*p* < 0.001) symptoms (Table 2). Mild–moderate and severe–extreme anxiety symptoms were more pronounced in RPL cases (*p* = 0.003). Similarly, mild–moderate and severe–extreme depression symptoms were more pronounced in RPL cases (*p* < 0.001). On the other hand, stress cases were in the mild–moderate category, and were comparable between cases and controls (*p* = 0.773), (Table 2).

**Table 3:** Multivariable linear regression models of stress, depression, and anxiety levels compared between women from case and control groups.

	DASS-21 Stress		DASS-21 Anxiety		DASS-21 Depression	
Variable	Coefficient (95% CI)	*p*	Coefficient (95% CI)	*p*	Coefficient (95% CI)	*p*
No RPL history	Reference		Reference		Reference	
RPL history	3.67 (2.00; 5.35)	<0.001	3.66 (2.21; 5.10)	<0.001	3.81 (2.25; 5.37)	<0.001
Age	0.02 (−0.10; 0.14)	0.73	0.02 (−0.08; 0.12)	0.66	0.00 (−0.11; 0.11)	0.99
BMI < 25	Reference		Reference		Reference	
BMI ≥ 25	1.02 (−0.66; 2.74)	0.24	0.83 (−0.64; 2.29)	0.27	1.26 (−0.32; 2.85)	0.12
Elementary education	Reference		Reference		Reference	
Secondary education	−1.57 (−4.31; 1.17)	0.56 *	−1.36 (−3.72; 1.00)	0.52 *	−1.05 (−3.60; 1.50)	0.84 *
University education	−1.44 (−3.51; 0.63)	0.36 *	−1.19 (−2.98; 0.59)	0.38 *	−1.75 (−3.68; 0.17)	0.16 *
Ethnicity		0.78		0.82		
Kazakhs	Reference					
Others	0.41 (−2.45; 3.27)		0.28 (−2.18; 2.74)			

* Corrected for multiple pairwise comparisons using the Bonferroni correction approach.

## Data Availability

All data related to this study are available from the authors (gulzhanat.aimagambetova@nu.edu.kz) upon reasonable request and with permission of NU IREC.
